# A subacute model of glaucoma based on limbal plexus cautery in pigmented rats

**DOI:** 10.1038/s41598-019-52500-2

**Published:** 2019-11-08

**Authors:** Rafael Lani, Mariana S. Dias, Carla Andreia Abreu, Victor G. Araújo, Thais Gonçalo, Gabriel Nascimento-dos-Santos, Adalmir Morterá Dantas, Silvana Allodi, Mario Fiorani, Hilda Petrs-Silva, Rafael Linden

**Affiliations:** 1 Instituto de Biofísica Carlos Chagas Filho, Universidade Federal do Rio de Janeiro, Rio de Janeiro, Brazil; 2 Faculdade de Medicina, Universidade Federal do Rio de Janeiro, Rio de Janeiro, Brazil

**Keywords:** Cell death in the nervous system, Retina

## Abstract

Glaucoma is a neurodegenerative disorder characterized by the progressive functional impairment and degeneration of the retinal ganglion cells (RGCs) and their axons, and is the leading cause of irreversible blindness worldwide. Current management of glaucoma is based on reduction of high intraocular pressure (IOP), one of its most consistent risk factors, but the disease proceeds in almost half of the patients despite such treatments. Several experimental models of glaucoma have been developed in rodents, most of which present shortcomings such as high surgical invasiveness, slow learning curves, damage to the transparency of the optic media which prevents adequate functional assessment, and variable results. Here we describe a novel and simple method to induce ocular hypertension in pigmented rats, based on low-temperature cauterization of the whole circumference of the limbal vascular plexus, a major component of aqueous humor drainage and easily accessible for surgical procedures. This simple, low-cost and efficient method produced a reproducible subacute ocular hypertension with full clinical recovery, followed by a steady loss of retinal ganglion cells and optic axons, accompanied by functional changes detected both by electrophysiological and behavioral methods.

## Introduction

Glaucoma, the leading cause of irreversible blindness worldwide, comprises a heterogeneous group of neurodegenerative disorders defined by the progressive functional impairment and death of the retinal ganglion cells (RGCs) and their axons^1,2^. A recent study estimates that approximately 76.0 million and 111.8 million people will be affected by glaucoma in 2020 and 2040, respectively^3^. Current therapeutic approaches are based on either clinical or surgical procedures to reduce high levels of intraocular pressure (IOP), one of the most consistent risk factors for the development and progression of glaucoma. However, approximately 45% of patients do not respond to these approaches and experience disease progression^4^. Therefore, understanding the complex, multifactorial pathophysiological features of glaucomatous neurodegeneration is required for the development of effective therapies to prevent or slow down the degenerative process, either independent of or in addition to lowering the IOP.

Modeling glaucoma in laboratory animals is a well-established strategy to both identify disease mechanisms and develop new neuroprotective approaches. In particular, rodents are low-cost, easy to handle, have a short life-span, can be genetically manipulated and the anatomy and physiology of their ocular structures are relatively similar to humans^5–8^. Among several distinct rodent models of glaucoma, those based on either acute or chronic ocular hypertension (OHT) most closely resemble disease in humans. Spontaneous OHT has been well characterized in mouse lines such as the DBA/2 J and DBA/2NNia strains, often used as models of age-related glaucoma^9^. However, these mice develop the disease relatively late in life, which demands a significant time commitment for the experiments^7^. There is also considerable variability in the course of both the rise of IOP and glaucomatous damage among animals of the same age^10^ from distinct laboratory facilities. In addition, a recent longitudinal study of 118 DBA mice described problems related to the measurement of the IOP, functional evaluation and *in vivo* retinal imaging, due to complex anterior segment pathology^11^.

Experimentally induced, unilateral OHT carries advantages over genetic models of glaucoma, such as better control over the optic nerve and retinal pathology, as well as the availability of the fellow eye as an internal control. In addition, the bigger size of the eye globe and related anatomic structures in rats, as compared with mice, facilitates surgically induced OHT. Here we describe a novel and simple method to induce ocular hypertension in pigmented rats, based on low-temperature cauterization of the whole circumference of the limbal plexus, a vascular component of the post trabecular pathway of aqueous humor drainage, immediately upstream to the episcleral veins. Full-circle limbal plexus cautery (LPC) induced subacute OHT, with progressive glaucomatous neurodegeneration that extends beyond the period of elevated IOP. This method is easy to perform, low-cost, efficient, and leads to the loss of ganglion cells within a relatively short time after a single surgical procedure.

## Results

### Surgery and clinical evolution

**Figure 1:**
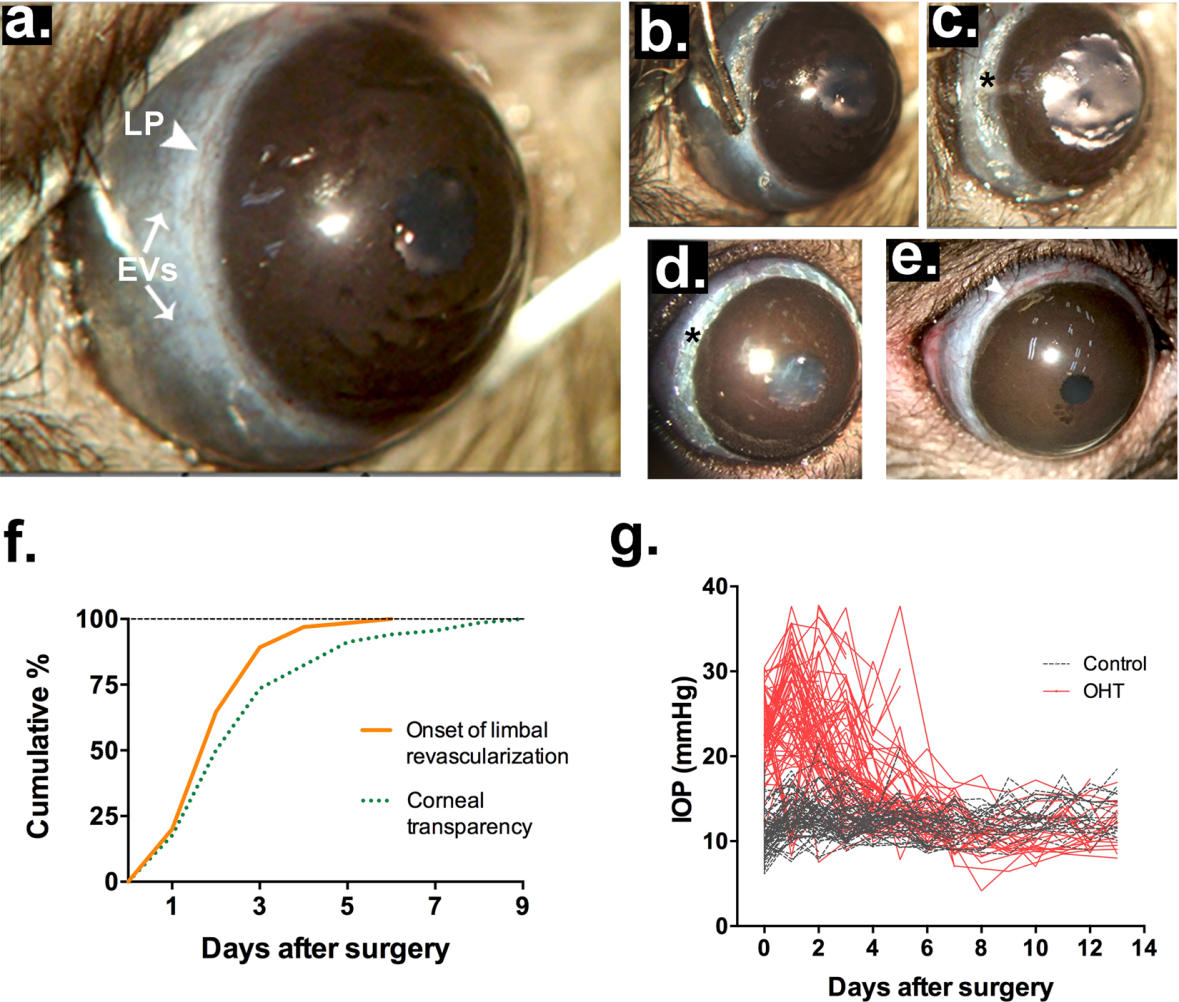
Limbal plexus cauterization and clinical evolution. (**a**) Pre-op, highlighting the limbal plexus (arrowhead) and a temporal episcleral vein (arrows); (**b**) Tip of the cautery applied on the limbal plexus. (**c**,**d**) Immediately post-op in two cases, highlighting the contiguous cauterization marks (asterisk) and either moderate (**c**) or no mydriasis (**d**). (**e**) Post-operative limbal revascularization. Images (**a**–**c**) were acquired during the procedure in the same animal, while (**d**,**e**) were taken from distinct animals. (**f**) Clinical recovery, expressed as the cumulative percentage of animals with signs of limbal revascularization (n = 65) and complete corneal transparency (n = 68) along the post-op period. (**g**) Time course of IOP measurements in OHT and control eyes (n = 69).

In pigmented rats, access to the limbal plexus from the surface of the globe is noninvasive (Fig. 1a). Following the measurement of basal IOP, the limbal vasculature of the right eye was cauterized by softly touching the vessels with the round tip of a low-temperature cautery (Fig. 1b), progressively along the whole circumference of the limbus, which leaves a continuous, conspicuous mark (Fig. 1c,d). Clinical follow-up and IOP measurements started immediately after surgery and were repeated daily, including standard post-op medication (see Supplementary Tables S1, S2). The onset of limbal revascularization was detected as early as the first 2 days after surgery (D1–2) in almost half of the operated animals (Fig. 1f), and all rats had started this process on the sixth day (D6). Corneal edema and diffuse opacification were often seen immediately after the procedure (Fig. 1e), but had subsided in 73.5% and 95.6% of animals at 3 and 7 days, respectively after surgery (Fig. 1f). At 14 days after surgery, 91.5% of the operated animals had recovered completely from the procedure and presented clinically normal eyes. A summary of clinical intercurrences is listed in Supplementary Table S3.

Cauterization of the limbal plexus induced transient ocular hypertension (Fig. 1g, Supplementary Fig. S1, Supplementary Table S2), characterized by an immediate rise of the IOP (22.5 ± 0.5 mmHg OHT vs. 10.7 ± 0.3 mmHg Control), with a peak at 1 day after surgery (24.8 ± 0.9 mmHg OHT vs. 12.9 ± 0.3 mmHg Control), and progressive return to normal values at D7 (12.0 ± 0.5 OHT × 11.8 ± 0.3 mmHg Control). The ratio of the IOP in operated and control eyes peaked at 2.2 ± 0.1 immediately after surgery, coincident with a small drop in the IOP of the control eye (see Supplementary Fig. S1). In only 6 out of 75 operated animals, the procedure failed to increase IOP, a success rate of 92.0% (see Supplementary Table S3).

### Loss of retinal ganglion cells

**Figure 2:**
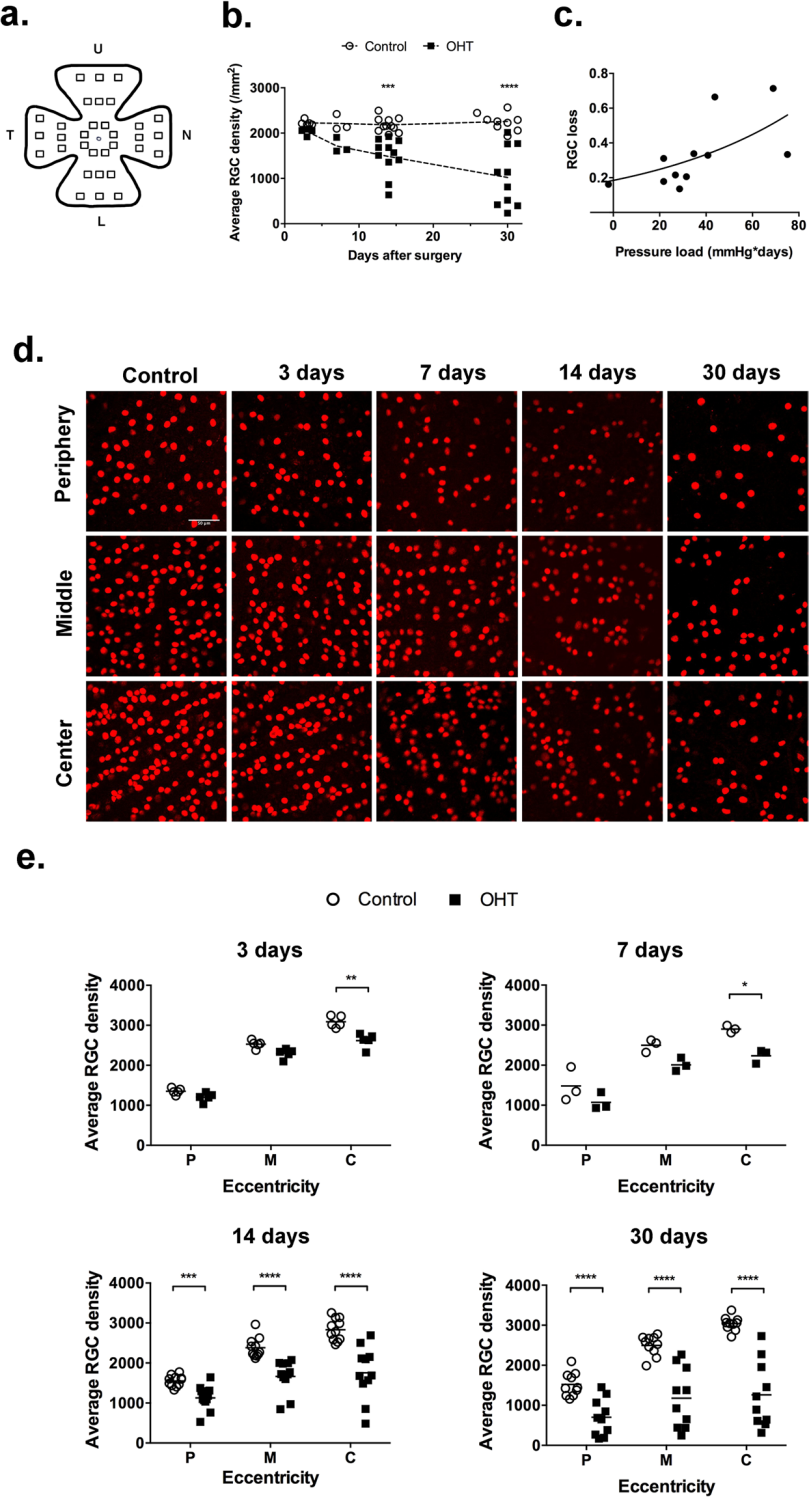
Limbal plexus cauterization induces RGC loss. (**a**) Scheme of a retinal whole mount, showing the regions of imaging acquisition for RGC counting. (**b**) Progressive RGC loss following subacute ocular hypertension (**c**) Correlation of RGC loss with pressure load at D14; Pearson correlation, r = 0.6464, p = 0.0316 (n = 11). (**d**,**e**) Regional distribution of RGC density at various times after the procedure. Statistical analysis (b, d and e): Two-way ANOVA followed by Sidak’s multiple comparisons test; (*) p < 0.05; (**)p < 0.01; (***)p < 0.001; (****)p < 0.0001.

**Figure 3:**
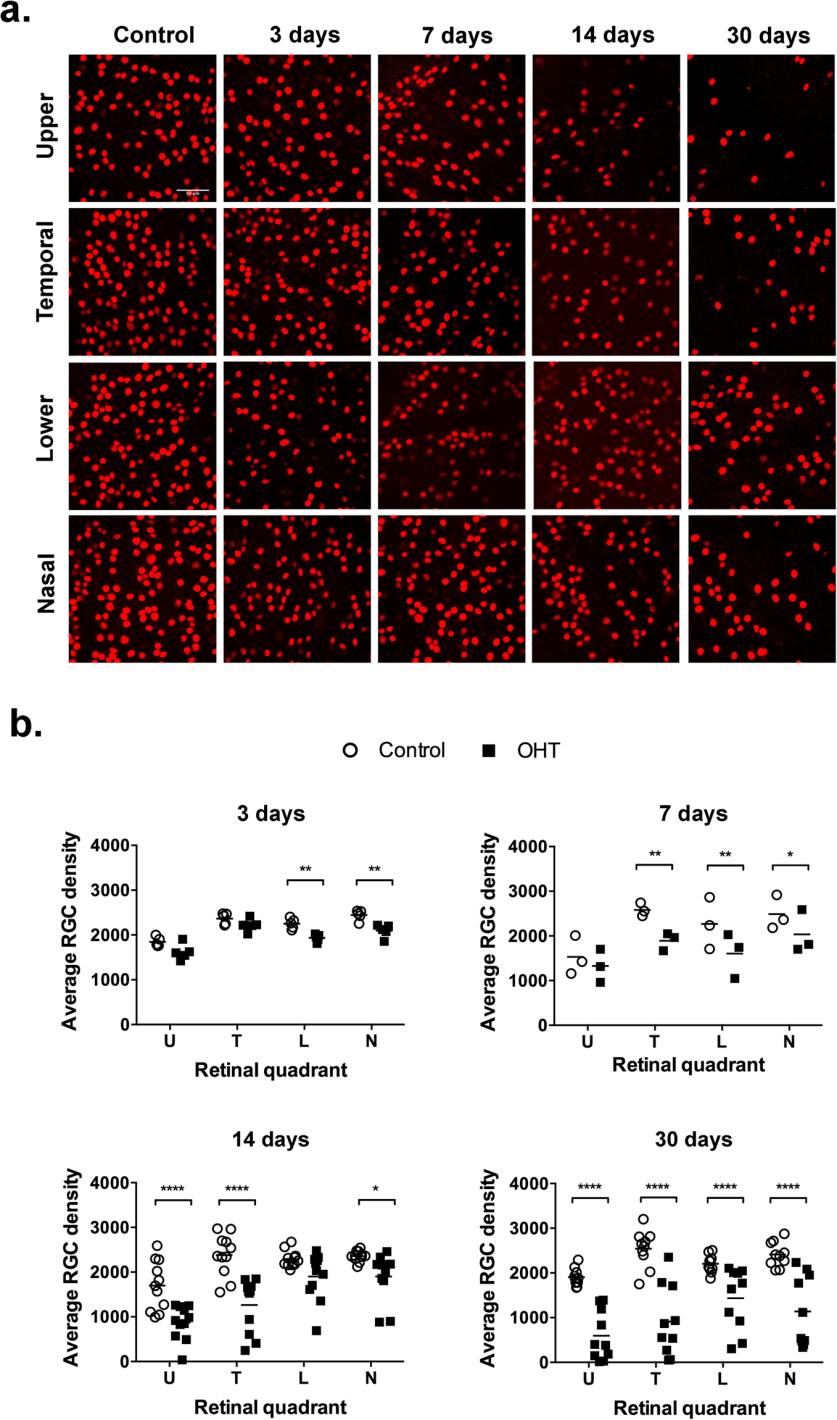
Regional loss of RGC. (**a**) Photomicrographs of representative counting fields of RGCs labeled with an antibody to Brn3a. (**b**) Distribution of average RGC density in the four quadrants of the retina. The graphs show the means (horizontal bar), and individual averages of RGC densities for 3–9 animals per time after the procedure. Statistical analysis: Two-way ANOVA followed by Sidak’s multiple comparisons test; (*)p < 0.05; (**)p < 0.01; (***)p < 0.001; (****)p < 0.0001.

The RGCs were identified in flat-mounted retinas as profiles immunostained with antibodies to the POU domain transcription factor Brn3a^12,13^ (Fig. 2a,d). Counts were done at 3, 7, 14 and 30 days after surgery, and RGC distribution was plotted for differing eccentricities (center to periphery, Fig. 2d,e) and quadrants (upper, lower, temporal and nasal, Fig. 3a,b). OHT led to a progressive reduction of the whole-retina average density of labeled RGCs as compared with control eyes (Fig. 2b). RGC loss was correlated with pressure load, i.e. the cumulative exposure to excess IOP from surgery to D14 (Fig. 2c). At D3, the peripheral and middle portions of the retina were still spared, while a mild decrease of about 15% of RGC density was detected in the central retina (Fig. 2d,e). Later on, RGC loss progressively increased in the central retina, and was also detected in the middle and peripheral regions (Fig. 2d,f–h).

Differential RGC loss was also detected when comparing retinal quadrants, with an early decrease of RGC density in both nasal and lower quadrants at D3, spreading to the temporal retina at D7 (Fig. 3a,b). At two weeks after OHT, RGC loss reached more than 50% at the upper and temporal quadrants but remained at about 20% in both nasal and lower quadrants (Fig. 3). RGC loss progressed to the whole retina at D30, reaching up to 70% in the upper and temporal quadrants (Fig. 3).

### Astrocytic response in the inner retina

**Figure 4:**
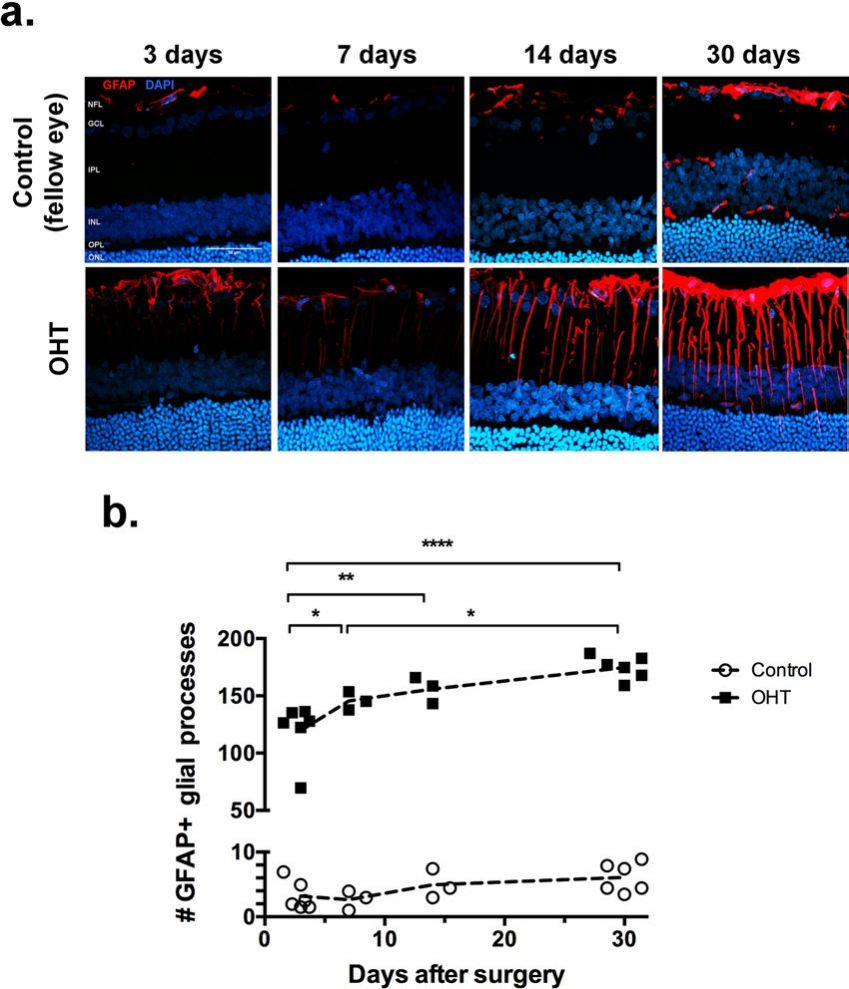
Progressive neuroinflammatory response to increased IOP. (**a**) Retinal sections immunostained for GFAP (red), and counterstained with DAPI (blue). (**b**) Numbers of vertical GFAP-labeled processes across the inner nuclear layer in sections from either control or operated eyes. NFL: nerve fiber layer; GCL: ganglion cell layer; IPL: inner plexiform layer; INL: inner nuclear layer; OPL: outer plexiform layer; ONL: outer nuclear layer. Statistical analysis: Two-way ANOVA followed by Sidak’s multiple comparisons test; (*)p < 0.05; (**)p < 0.01; (***)p < 0.001; (****)p < 0.0001.

Glial Fibrillary Acidic Protein (GFAP) is a cytoskeletal protein widely used as a marker of reactive changes in astrocytes and Müller glial cells^14^, which is one of the hallmarks of inflammatory responses in the retina. We found a progressive increase in the number of GFAP^+^ cell processes across the inner plexiform layer of the retina (Fig. 4).

### Loss of optic nerve axons

**Figure 5:**
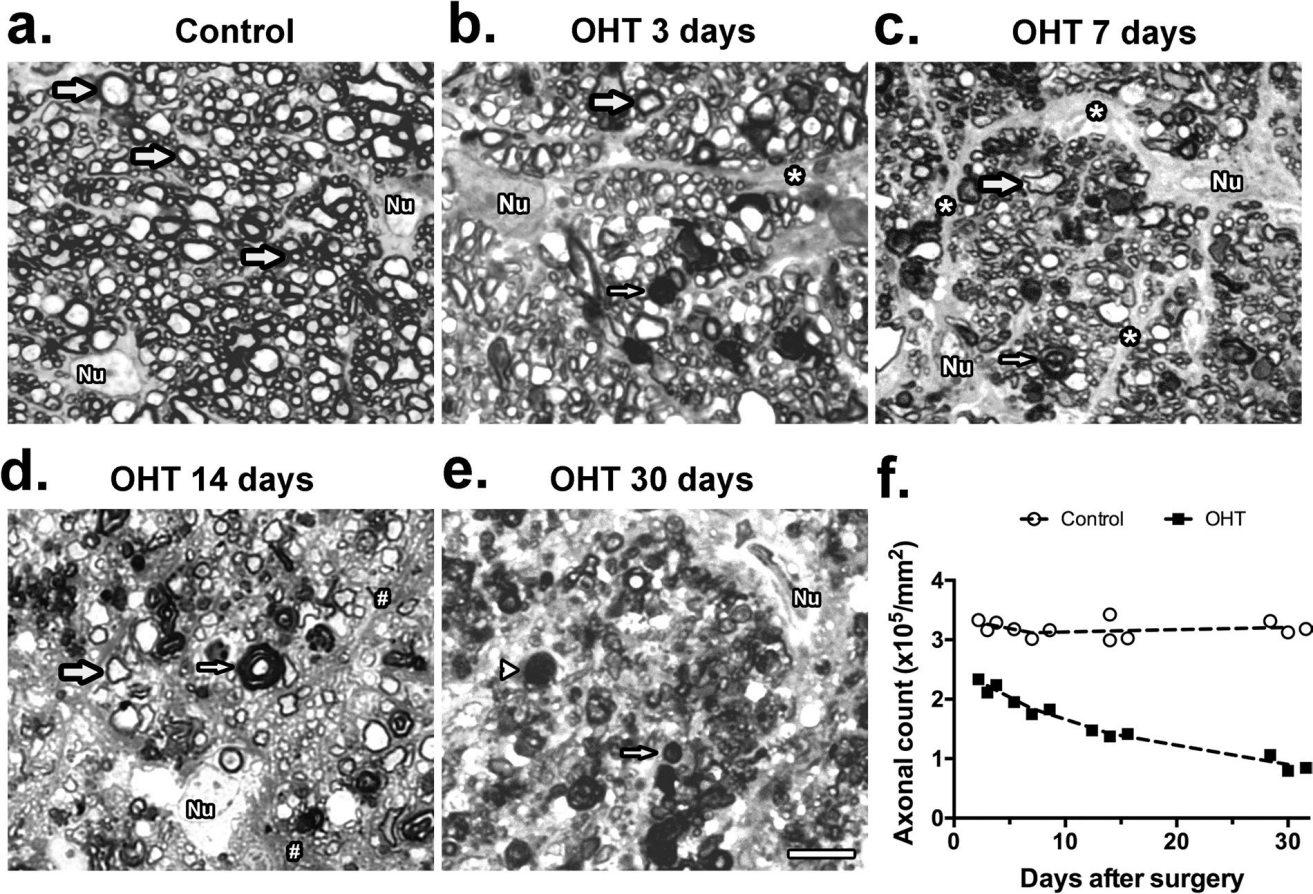
Markers of optic nerve degeneration following OHT. (**a**–**e**) Representative photomicrographs of optic nerve sections from (**a**) normal optic nerve, and (**b**–**e**) at 3, 7, 14, and 30 days after the limbal cauterization (OHT). Thick arrows: normally myelinated axons; thin arrows: dark-degenerating axons; asterisks: hypertrophic glial processes; hashes: vacuolar degeneration of glial cell process; arrowheads: fragmentation of myelin. (**f**) Axon counts at distinct times after OHT. Two-way ANOVA followed by Sidak’s multiple comparisons test resulted in p < 0.0001 in control x OHT groups at all time points. Nu = Nucleus.

As a preliminary assessment of optic nerve damage following LPC, we examined RGC axons in toluidine blue-stained semithin sections, at approximately 1 mm from the posterior pole of the eye (Fig. 5a–e). Photomicrographs of control optic nerves exhibited fibers of normal aspect, various sizes, with a smooth outline (Fig. 5a). At D3, a small number of profiles contained dense, amorphous material typical of dark degeneration, and hypertrophy of glial cell processes was visible among normal axons (Fig. 5b). At D7, the most striking change was an increased disorganization of the nerve fibers, due to the invasion of glial cell processes among axons with signs of degeneration (Fig. 5c). At D14 there was a marked decrease in the density of normal axon profiles, some fibers showed detachment and vacuolization of myelin, and glial cell processes displayed vacuolar degeneration (Fig. 5d). Signs of degeneration were even more clear at D30, when some fibers showed extensive fragmentation of myelin, and degenerating axons were abundant (Fig. 5e). The number of normal-looking axons decreased steadily with time after limbal cauterization (Fig. 5f).

### Changes in the electroretinogram

**Figure 6:**
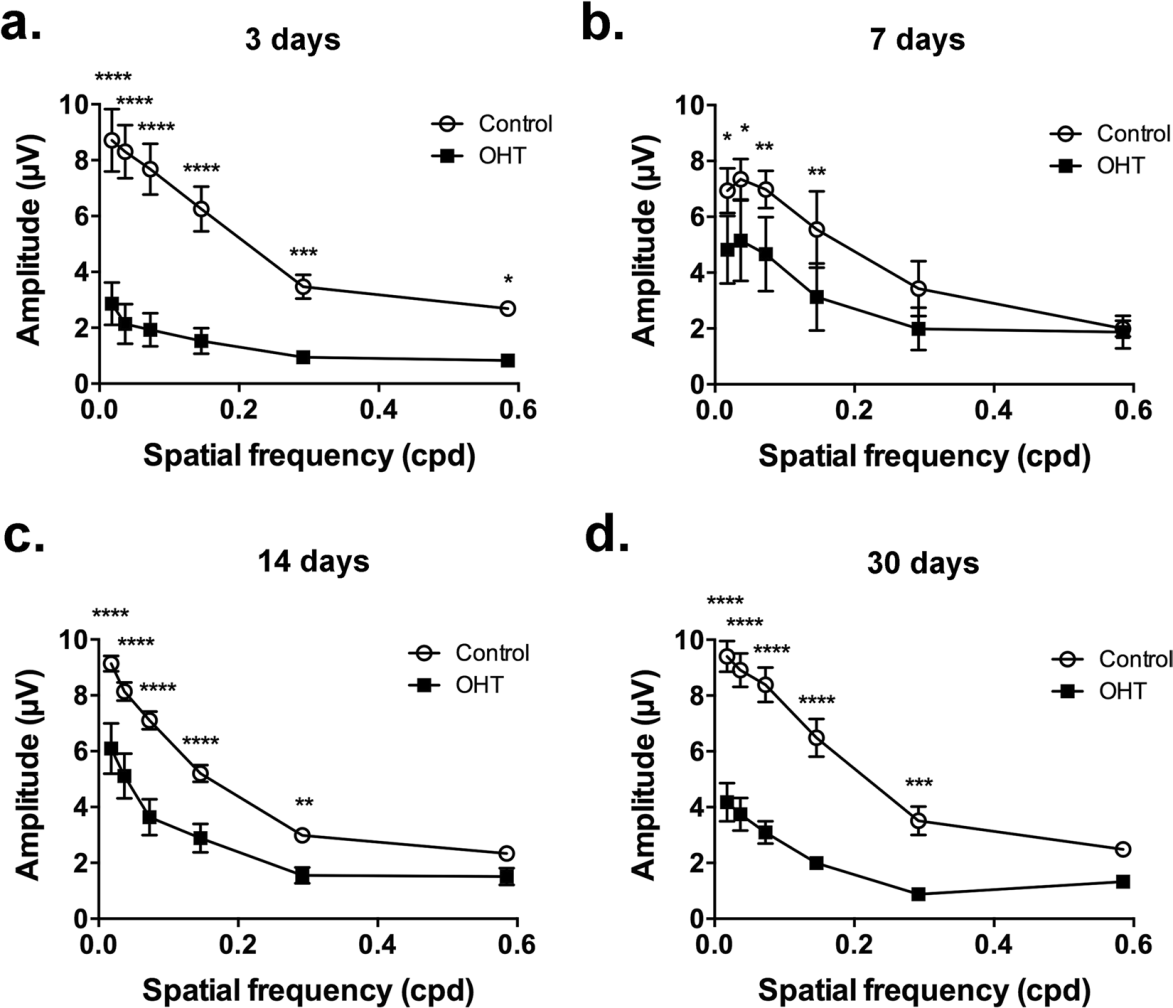
Pattern-ERG. Graphs show mean ± SEM amplitude of steady-state PERG at (**a**) 3, (**b**) 7, (**c**) 14, and (**d**) 30 days after surgery. Two-way ANOVA followed by Sidak’s multiple comparisons showed differences between control and OHT groups at (*)p < 0.05; (**)p < 0.01; (***)p < 0.001; and (****)p < 0.0001. Cpd = cycles per degree.

The high rate of quick clinical recovery allowed *in vivo* functional evaluation of the retina in most operated animals. Pattern electroretinogram (PERG) has been traditionally used both clinically and experimentally as an effective and noninvasive test to study RGC function^15^. As early as D3, when IOP was still elevated but only a mild and localized RGC loss was observed in the retina, there was a robust lowering of the amplitude of the steady-state PERG in experimental eyes compared with their respective controls (Fig. 6). Interestingly, the difference in PERG amplitude between OHT and control eyes appeared to recede at D7, followed by a progressive increase at D14 and D30.

Flash electroretinogram (FERG)^16^ was also recorded in both photopic and scotopic conditions (see Supplementary Fig. S2). The amplitude of the FERG response followed a similar profile as seen in PERG, consistent with impaired function of the outer retina at the period of ocular hypertension (D3), followed by a return of FERG values to control levels at D7 and D14, and decreased responses at D30 (see Supplementary Fig. S2).

### Visuobehavioral impairment

**Figure 7:**
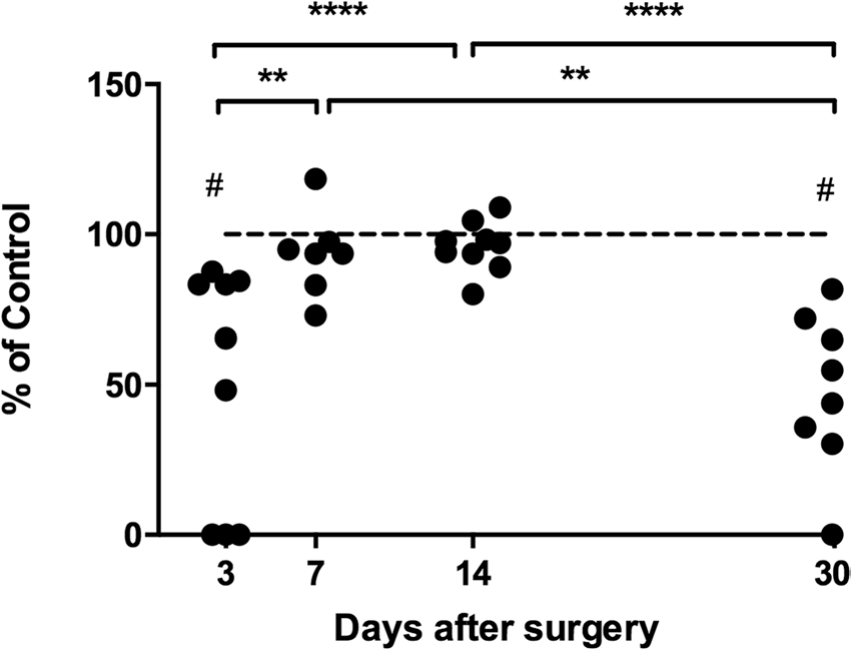
Optomotor responses. Data expressed as % of baseline-corrected control. Two-way ANOVA followed by Sidak’s multiple comparison test results indicated by (**)p < 0.01; (****)p < 0.0001 among animals at various times after LHC, (#)p < 0.0001 between OHT and control.

To examine how LPC-induced OHT affects rat visual behavior, the optomotor response (OMR) was examined through observation of the animal’s ability to track a moving patterned visual stimulus (alternate, vertical black and white grids) at various spatial frequencies. The visual threshold at distinct intervals following OHT showed a profile similar to that of electrophysiological tests, with reduced visuomotor acuity at D3, then a return to baseline levels at D7 and D14, and a significant decrease at D30 (Fig. 7).

## Discussion

Widely used experimental models of glaucoma have been classified as pre-trabecular, trabecular or post-trabecular, using the trabecular meshwork (TM) as an anatomic reference^17^. Multiple limitations have, however, been reported: (1) intracameral injection of viscoelastic substances or microbead particles (pre-trabecular)^18,19^ is invasive, with high risk of corneal damage or inflammation, as well as intraocular infections; moreover, some injected substances may reduce the transparency of the optic media, which precludes functional analysis; (2) laser scarring of the TM and/or photocoagulation of the limbal vasculature and episcleral veins (trabecular)^20,21^ require expensive equipment, are difficult to apply to pigmented animals due to high and variable absorption of laser energy, and is often accompanied by damage to the TM, corneal inflammation and ulceration, which impair functional assessment^22^; (3) Morrison’s procedure of injection of hypertonic saline into an episcleral vein (post-trabecular)^23^ requires extensive training, and leads to highly variable IOP^7,24^; (4) Shareef and Sharma’s episcleral vein cauterization (EVC)^25^ is also invasive, requires a slow learning curve to access the deep episcleral veins behind the rectus muscles, may lead to venous congestion because the targeted deep venous plexus actually comprises the vortex veins, which drain the entire globe including the choroid^26,27^, and leads to highly variable period of OHT, ranging from 2 weeks to 6–7 months as described by differing authors^25,28,29^.

The currently described method of limbal plexus cauterization (LPC) leads to a post-trabecular type of ocular hypertension, followed by retinal and optic nerve degeneration. This model is akin to human glaucoma secondary to increased drainage pressure of the aqueous distally to the trabecular meshwork, as seen in cases of Grave’s orbitopathy, cavernous sinus thrombosis, retrobulbar tumors and arterio-venous shunts^30^. The subacute elevation of the IOP was followed by clinical recovery in about 90% of the rats and enabled the functional evaluation of the retinas *in vivo* as early as the third day after surgery in roughly three-quarters of the operated animals. The LPC procedure has several advantages over other methods: (1) it is technically easy and noninvasive, with a quick learning curve; (2) it does not require expensive equipment; (3) it allows both *in vivo* electrophysiological and behavioral examination; (4) the reproducibility and success in generating glaucoma reduces the numbers of animals necessary for experiments, thus complying with increasing ethical concerns regarding the use of animals in basic research. Notwithstanding, structural damage in the ganglion cell layer was detected with relatively short latency, with loss at two weeks of about one third, and at one month of about half of the retinal ganglion cells, as well as extensive axon loss at the optic nerve. Therefore, it allows for studies of glaucomatous neurodegeneration, following either short- or mid-term experimental designs.

LPC all around the cornea induced immediate OHT, which peaked at 1–3 days after surgery (Fig. 1) and progressively decreased to basal levels within one week. Although most cases of human glaucoma are classified as primary open-angle glaucoma, commonly associated with chronic ocular hypertension^1,31^, many such patients undergo progressive visual loss despite adequate control of IOP with either topical hypotensive drugs or surgical approaches^4,32,33^. Gradual worsening of visual function and further structural loss in these cases most likely reflects secondary degeneration dissociated from ocular hypertension. Thus, the present subacute OHT model may contribute to a better understanding of the glaucomatous neurodegeneration to the benefit of patients^34^. The LPC-induced immediate IOP peak, to about twice the control value, is also akin to an acute angle closure crisis, marked by sudden IOP rise due to either complete or near complete anatomical blockade of the iridocorneal angle^35^, and the LPC model may help understand mechanisms of retinal degeneration due to the latter condition.

Structural damage followed a regionalized pattern in the retina. Both the prevalence of degeneration in the central retina (distant from the limbus), as well as the significant correlation between cell loss and pressure load (Fig. 3c), rule out the attribution of RGC loss to direct heat-induced damage. On the other hand, the higher rates of cell loss in the upper and temporal quadrants are similar to other rodents OHT models^36–38^. Progressive and regionalized visual field loss is typical of human glaucoma, but in humans the lower temporal quadrant and peripheral retina tend to be the most sensitive to IOP damage, differing from the pattern observed in our pigmented rat^31^. It is possible that the differences are associated with histological features of the optic nerve head, which in the rat has a glial lamina cribrosa and a peculiar pattern of lamina strain and peripapillary sclera deformation upon pressure^39,40^.

The early appearance and progressive increase in GFAP immunostaining of glial cell processes, support the hypothesis that neuroinflammation is an early event of the pathophysiology of glaucoma^41–44^, as shown in other animal models^45–47^ as well as in human patients^48,49^. Nevertheless, controversies about the actual contribution to glaucoma of neuroinflammatory responses, inclusive of other glial cell types, are still unsettled^49,50^.

As soon as 3 days after LPC, the steady-state PERG recordings indicated an impairment of electrophysiological responses. At that time, experimental eyes were hypertensive and the numbers of normal looking axons were only slightly reduced, but RGC numbers were similar in both eyes. Following the return of IOP to basal levels at 7 days after LPC, PERG amplitude recovered slightly but remained significantly lower than in the fellow eyes. From this time onwards, the numbers of both Brn3a-labeled RGCs and optic axons, as well as PERG amplitude declined in synchrony.

The time course of PERG changes, as well as those of both FERG and OMR, suggest an immediate functional effect directly tied to increased OHT, followed by partial recovery and progressive functional loss dissociated from the subacute insult. It has been proposed that increased IOP, even without an established cell loss, is able to impair retinal function both in humans and in animal models of glaucoma^16,51–54^. Moreover, decreased IOP in ocular hypertensive patients and mammals is associated with variable degrees of functional recovery^55–57^. Functional improvement associated with recovery of normal IOP levels may also be related to a recently characterized axogenic mechanism that produces transient enhancement of the excitability of mouse ganglion cells after induction of OHT^58^.

Finally, the preservation of normal optomotor tracking reflex at both D7 and D14 whereas PERG amplitude was reduced, is similar to findings in transgenic models of photoreceptor degeneration^59^. The optomotor response is a compensatory reflex that requires functional communication between ON-OFF direction-selective RGCs and the nucleus of the optic tract, and has been widely used as a sensitive test to study visual function in rodents^60^. Given the selective involvement of a minor population of RGCs, as well as their restricted axonal projections, our result suggests that retinal damage caused by subacute OHT may also show cell-type specificity^34,61,62^. Future studies are required to confirm this hypothesis. Notably, an impact of ocular hypertension upon blood flow dynamics at the optic nerve head has been suggested as a major mechanism of initiation of glaucomatous damage, with ensuing compromise of RGCs energy metabolism^63^, and oxidative stress markers have been found in the serum, retina and aqueous humor of glaucoma patients^64^. Notwithstanding, distinct models of ocular hypertension that led to IOP levels similar to our study failed to find vascular changes or hypoxic damage^25,65^, and retinal ischemia caused by ocular hypertension was reported only at IOP levels varying from 110 mmHg to 140 mmHg^7,66,67^. Given that a threshold level of IOP required to induce retinal ischemia in rats has been estimated at higher than 60 mmHg^68,69^, even considering the depressing effect of general anesthesia^70^, the current average peak IOP of 24.8 ± 0.9 mmHg at post operative day one (Supplementary Fig. S1a) is lower than expected for the doses of ketamine and xylazine used in our study, when compared with the IOP depressing effect of about 50% for much higher doses of the same anesthetics^70^. Thus, it is unlikely that the degenerative effects in the present study are due to robust ischemic events, although subtle changes in blood flow at the optic nerve head cannot be discarded.

Also, the subacute IOP rise characterized in our model is transient and admittedly does not mimic the most common features of chronic glaucomatous disease progression. Nonetheless, the current procedure provides a relatively easy, robust and reproducible model of the disease, by combining a cardinal modifiable risk factor with both defining morphological and functional neurodegenerative markers, and therefore may contribute to both the disclosure of cellular and molecular mechanisms, as well as the testing of novel therapies applicable to secondary degeneration in glaucoma.

## Conclusion

Ocular hypertension following full-circle limbal plexus cautery constitutes an easy and reproducible model of glaucoma in pigmented rats. The model is characterized by a subacute increase of the IOP that leads to progressive structural damage to the inner retina and optic nerve with functional consequences. This model provides a unique post-operative clinical recovery rate, which facilitates *in vivo* functional studies and may contribute both to better understand the pathophysiology of glaucoma, as well as to develop novel therapeutic approaches to glaucomatous neurodegeneration.

## Materials and Methods

### Animals

A total of 69 pigmented Lister Hooded rats (male and female; aged 2–3 months; 180–320 g) were handled following the recommendations from the Association for Research in Vision and Ophthalmology (ARVO) Statement for the Use of Animals in Ophthalmic and Vision Research. The experimental protocols were approved by the Ethics Committee on the Use of Animals in Scientific Experimentation from The Health Sciences Center, Federal University of Rio de Janeiro (protocol 083/17). The animals were housed in controlled conditions of temperature and 12-hr light-dark cycle (lights on at 6 am and off at 6 pm), with standard food and water *ad libitum*.

### Induction of OHT

Rats were deeply anesthetized with an intramuscular injection of a solution containing ketamine hydrochloride (75 mg/kg) and xylazine hydrochloride (5 mg/kg). After 5 minutes, the surgical plane of anesthesia was checked by toe pinch, the right eyelids were kept separated with the aid of a curved forceps, and the globe was gently pushed forward to help expose the limbal vasculature and allow topical anesthesia of the eye surface with ophthalmic proxymetacaine hydrochloride 0.5%. The animal was placed on slight lateral decubitus, and a low-temperature ophthalmic cautery (Bovie Medical Corporation, Clearwater, FL) was used to cauterize the limbal plexus in a 360° approach around the cornea, visualized through a stereo microscope (Carl Zeiss, Germany) at 40x magnification. Care was taken not to compromise the periphery of the cornea. Clinical signs of a successful procedure were the presence of small circular cauterization marks on the scleral limbus, the disappearance of blood along the limbal vasculature, and mydriasis in the experimental eye. After surgery, a drop of ophthalmic prednisolone acetate (10 mg/mL) was applied, and maintained in contact with the anterior surface of the right eye during approximately 40 seconds, after which it was replaced by an ophthalmic ointment of antibiotics (oxytetracycline hydrochloride 30 mg/g and polymyxin B 10.000 U/g).

### Measurement of IOP and clinical follow-up

Rats were lightly anesthetized (ketamine: 18,75 mg/kg; xylazine: 1,25 mg/kg), and IOP was measured using a handheld tonometer (Tono-Pen^®^ XL, Reichert, Buffalo, NY)^71^ applied to the surface of the eye topically anesthetized with ophthalmic proxymetacaine hydrochloride 0.5%. The tip of tonometer touched the center of the cornea perpendicularly, just enough to move the globe slightly posterior^26^. Measurements were done immediately before and after the surgical procedure, then every day in the morning up to 14 days after surgery. An average of 12 to 20 individual readings were taken from each eye, possible “off” readings and those that resulted in instrument-generated averages with >10% error^71^ were discarded. The experimental eyes were examined under a magnifier, and clinical evolution was recorded of corneal transparency, limbal revascularization, chemosis and conjunctival hyperemia, or other intercurrences such as hyphema, corneal dystrophy or scleral instability associated with uveal prolapse.

### Electroretinography (ERG)

Both the flash electroretinogram (FERG) and the pattern electroretinogram (PERG) were recorded at 3, 7, 14 and 30 days after surgery. The animals were deeply anesthetized with intramuscular ketamine and xylazine (75 mg/kg and 5 mg/kg, respectively), so as to decrease the chances of artifacts through involuntary muscle movements and other sources of noise during the exam. An additional injection of one half the initial dose was usually necessary during the exam, to maintain the animals properly anesthetized throughout the ERG assessment, which lasted approximately 90 minutes. The anesthetic agents used do not affect the amplitude of the responses^72,73^. The cornea was topically anesthetized with a drop of proxymetacaine hydrochloride 0.5% (Anestalcon^®^; Novartis Biociências S.A., São Paulo, SP), and the active electrode (a stainless steel needle 0.25 mm × 15 mm; Hansol Medical Co, Korea) was inserted carefully so as to avoid piercing the cornea, at the temporal periphery of the transitional zone^74^. The reference and ground electrodes were also stainless steel needles (0.4 mm × 37 mm; Chalgren Enterprises, USA), positioned subcutaneously into the ipsilateral temporal canthus and the left hind limb, respectively. Signals acquired with the recording electrode were differentially amplified, digitized and processed using a MEB-9400K system (Nihon Kohden Corporation, Japan). The waveforms were stored and analyzed using the software Neuropack Manager v08.33 (Nihon Kohden Corporation, Japan).

The full protocol was recorded sequentially for either eye, beginning with the right (experimental) eye, and consisted of the FERG to evaluate the function of the outer retina (photoreceptors and bipolar cells), followed by the PERG to assess the function of the RGCs. After certifying the surgical plane of anesthesia, and with the animals adapted to the environmental light (white; ~140 LUX), the photopic FERG was recorded using a white strobe flash (SLS-3100 flash stimulator, Nihon Kohden Corporation, Japan), with 10 ms duration and 20 J energy, generated by a xenon lamp at 20 cm from the stimulated eye, and 20 individual responses (1 Hz) were averaged. We analyzed the b-wave amplitude and implicit time, a component that reflects the function of the On-bipolar cells related to the cone pathway. The activity of cones was isolated through the flicker ERG, recorded after the photopic FERG with flash energy of 1.2 J and frequency of 15 Hz. Twenty individual responses were averaged, and the amplitude at the frequency of the stimulus was extracted off-line using a fast Fourier transform (FFT) algorithm programmed with Matlab software (The MathWorks, USA). Then, the rats were dark adapted for scotopic FERG recording. We recorded a 20 J single flash response, which elicited a mixed potential from both rods and cones (a-wave amplitude and implicit time) and their related On-bipolar cells (b-wave amplitude and implicit time), with a predominance of the rod pathway. We used a short scotopic protocol of 3 minutes in completely darkness, both to study the kinetics of retinal dark adaptation and to abbreviate the exam, as a measure to prevent the occurrence of cataract due to corneal damage induced by the active needle electrode. Signals were differentially amplified, digitized at a sampling interval of 100 µs, filtered (band-pass: 0.1 Hz–100 kHz; no notch filter was used) and stored for subsequent analysis.

The steady-state PERG amplitude gathers the component of the wave most likely associated with the RGC bioelectrical response (NII deflection of the transient-state PERG), generating a stable and reproducible sinusoid. The stimulus consisted of contrast reversal of a checkerboard with black and white squares alternating at 15 reversals/sec, with constant average luminance (250 cd/m^2^), presented at an LCD monitor (23″; model LS23B550, Samsung Electronics Co., Ltd., Korea) positioned at 20 cm from the examined eye. The band-pass filter was set to 1 Hz–100 Hz, and signals were averaged 200–300 times to stand out from noise. Six distinct spatial frequencies were presented, in the following order for all animals: 0.018; 0.585; 0.073; 0.037; 0.292; and 0.146 cycles/degree (cpd). The amplitude of the stored sinusoidal waveforms was analyzed using a custom-made FFT algorithm programmed through Matlab language (The MathWorks, USA).

### Optomotor response (OMR)

The animals were positioned in an Optomotry^®^ system^75^ (CerebralMechanics, Alberta, Canada), one at a time, on top of a platform in the middle of the arena delimited by 4 computer monitors arranged in a quadrangle. Before starting the exam, the rats were allowed to habituate for approximately 2 minutes on the platform. The test was done in photopic conditions, while the monitors displayed the image of a virtual cylinder with vertical alternate black and white stripes, moving around the animal in a fixed rotation speed (12 degrees/seconds) and contrast (100%). The rats were watched by one observer (RLa) through a video camera positioned above the platform. The experimenter followed the freely moving rat’s head with a crosshair superimposed on the video image, which corresponded to the center of the virtual cylinder^75^. The OMR is a smooth tracking movement of the animal’s head and neck in concert with the rotation direction of the gratings, of which clockwise or counter-clockwise movements test the left or the right OMR-related visual pathway, respectively. The spatial frequency of the stimulus (cycles/degrees) was incrementally changed until the tracking movement couldn’t be noticed by the experimenter anymore. The highest spatial frequency that elicited a clear OMR was considered the threshold of visual acuity of the tested eye. The Optomotry^®^ software was set on manual/separate mode, and each animal was examined in single time points (naïve testing)^59^. When an animal eventually dropped off the platform during the exam, it was immediately returned to the platform and the test was resumed^69^.

### Quantification of RGCs and labeling of astrocytes

The rats were euthanized by inhalation of carbon dioxide, the eyeballs were carefully removed and chemically fixed by immersion in 4% paraformaldehyde (PF4%) in phosphate buffer 0.1 M overnight at 4 °C. For RGC counting, retinas were dissected so that tissue topographic orientation was preserved, using the nasal caruncle and the choroid fissure as landmarks^36,76^. Then, the retinas were immunolabeled with an anti-Brn3a primary antibody (goat polyclonal IgG, 100 µg/ml, sc-31984, Santa Cruz Biotechnology; 1:1000 diluted in PBS 1x + BSA 0.1%), followed by a donkey anti-goat polyclonal IgG secondary antibody, conjugated with Alexa Fluor^®^ 555 (2 mg/ml, A21432, Thermo Fisher Scientific; 1:1000 diluted in PBS 1x+ BSA 0.1%), at RT. DAPI (4′,6-diamidino-2-phenylindole) was used for nuclear staining (10 minutes, at RT). The retinas were flat mounted in glass slides with the vitreous side up, and coverslipped in antifade mounting medium (DAKO; S3023; Dako Noth America, CA, USA).

To estimate the density of RGCs, flat mounts were examined under a confocal epifluorescence microscope (LSM 510 Meta, Zeiss) using a Plan-Neofluar 40x/1.3 objective. For each quadrant of the retina, 8 photos were taken, of which 2 were from central retina (~0.9 mm from optic disc), 3 from mid-retina (~2.0 mm from optic disc) and 3 from peripheral retina (~3.7 mm from optic disc), to a total of 32 photos per retina. Using the FIJI software^77^, Brn3a+ cells were counted by an experimenter unaware of the source of the photos, and mean cell density was estimated from these counts for each retina.

For immunolabeling of astrocytes, eyeballs fixed overnight with PF4% were cryoprotected by immersion in sacarose 30% overnight at 4 °C, embedded in Optimal Cutting Temperature Compound (O.C.T.™; Tissue-Tek^®^, 4583; Sakura Finetek USA), and frozen with liquid nitrogen. Cryostat sections (14 µm thick) from the temporal hemi-retina were mounted on gelatin-coated glass slides and immunostained by overnight incubation at 4 °C with anti-GFAP primary antibody (mouse monoclonal IgG, 200 µg/ml, sc-33673, Santa Cruz Biotechnology; 1:500 diluted in PBS-Triton x100 0.1% + BSA 1%), developed with donkey anti-mouse polyclonal IgG secondary antibody, conjugated with Alexa Fluor^®^ 555 (2 mg/ml; A31570, Thermo Fisher Scientific; 1:1000 diluted in PBS 1x and coverslipped in antifade mounting medium (DAKO; S3023; Dako Noth America, CA, USA). Sections were examined under an Axiovert 200 M (Zeiss) microscope with a 100x/1.3 objective. For each animal, GFAP^+^ processes crossing the inner plexiform layer were counted in a total of 9 fields from at least 3 distinct sections Representative images were acquired under a LSM 510 Meta (Zeiss) microscope using a Plan-Neofluar 40x/1.3 objective.

### Processing of the optic nerve for semithin sections

Following euthanasia and ocular enucleation, 2 mm-long segments of the optic nerve at 1 mm distal to the eye were fixed by immersion in 2.5% glutaraldehyde for 2 h, washed in 0.1 M cacodylate buffer (pH 7.4), and postfixed for 1 h in 1% osmium tetroxide containing 0.8% potassium ferrocyanide and 5 nM calcium chloride in 0.1 M cacodylate buffer (pH 7.4). Segments were rinsed in 0.1 M cacodylate buffer (pH 7.4) and distilled water, and block-stained in 1% uranyl acetate overnight, dehydrated in a graded acetone series, infiltrated with Polybed 812 resin (Polysciences) and polymerized at 60 °C for 48 h.

### Morphometric analysis of the optic nerve

Semithin (500 nm) cross sections were cut in an RMC ultramicrotome and collected on slides, stained with toluidine blue, mounted with Polybed 812 resin, and imaged under a Zeiss Axioskop 2 Plus microscope (Zeiss). For quantitative analyses, 4 images at 100x magnification were taken systematically from the cross sections of each nerve. Images were processed with the software ImageJ, and the number of myelinated fibers was manually counted by the same person (C.A.A.).

### Statistical treatment

Statistical analysis was performed using GraphPad Prism 6.0c software. Data are expressed as mean ± SEM, except for Fig. S1 that is expressed as mean ± 95% CI. Results with two independent variables were compared using two-way ANOVA, followed by Sidak’s post hoc test. P-values less than 0.05 were considered statistically significant.

## Supplementary information

Dataset 1

## Data Availability

All materials, data and protocols used in this research are available to be promptly shared with readers and the Editorial Board Members.
