# Supplementary material for: A subacute model of glaucoma based on limbal plexus cautery in pigmented rats
Source: Sci Rep. 2019 Nov 8;9:16286. doi: 10.1038/s41598-019-52500-2 (PMC6841973; doi:10.1038/s41598-019-52500-2)
Supplement: Supplementary file 1 — Dataset 1 [file 41598_2019_52500_MOESM1_ESM.pdf]

A subacute model of glaucoma based on limbal plexus cautery in pigmented rats

Rafael Lani[1], , Mariana S. Dias[1], Carla Andreia Abreu[1], Victor G. Araújo[1], Thaís Gonçalo[1], Gabriel Nascimento-dos-Santos[1], Adalmir Morterá Dantas[2], Silvana Allodi[1], Mario Fiorani[1], Hilda Petrs-Silva[1]*, Rafael Linden[1]*.

[1]Instituto de Biofisica Carlos Chagas Filho, Universidade Federal do Rio de Janeiro, Rio de Janeiro, Brazil.

[2]Faculdade de Medicina, Universidade Federal do Rio de Janeiro, Rio de Janeiro, Brazil.

*HPS and RLi contributed equally to this manuscript and should be considered co-senior authors

Correspondence should be addressed to: RLa (rafa.lani@biof.ufrj.br), HPS (hilda@biof.ufrj.br) or RLi (rlinden@biof.ufrj.br), at Instituto de Biofísica da UFRJ, Rio de Janeiro 21941- 901, Brazil. (rafa.lani@biof.ufrj.br, hilda@biof.ufrj.br, rlinden@biof.ufrj.br).

Drug	Regimen
Prednisolone Acetate, 10 mg/mL	Single dose (D0)
Ketorolac Trometamol, 5 mg/mL<br>Oxytetracycline Hydrochloride, 5mg/g<br>Polymyxin B, 10.000U/g	1x/day, D1-D5

Supplementary Table S1. Drugs and schedule during the postoperative period. D0: immediately after surgery; D1, D5: first, fifth days after surgery.

Days after surgery	OHT			Control		
	Mean	SEM	N	Mean	SEM	N
0						
Pré-op	12,38352	0,2844456	69	12,99485	0,2850212	69
Pós-op	22,49981	0,5145771	69	10,69535	0,2639739	69
1	24,77146	0,8544446	68	12,87408	0,2782238	68
2	23,12738	0,7316009	68	12,89513	0,2967597	68
3	20,13435	0,9030998	65	12,75369	0,2976713	65
4	16,96393	0,5898867	57	12,2315	0,1923575	57
5	15,86372	0,8175853	47	12,89482	0,3519894	47
6	13,21354	0,5191646	36	11,44581	0,3345537	36
7	11,96584	0,5002578	32	11,7946	0,3318546	32
8	10,44675	0,554732	21	11,95625	0,3818122	21
9	10,78061	0,5568793	19	12,62799	0,5326206	19
10	11,10291	0,5403543	21	12,83114	0,3246871	21
11	11,47046	0,5874162	17	13,19849	0,5900638	17
12	11,78146	0,6544889	18	13,13186	0,465199	18
13	12,17058	0,6233512	17	13,30498	0,5999621	17

Supplementary Table S2. IOP mean values with standard error (SEM) and the number of animals (N).

Procedure notes	Percentage
**IOP rise***	92.00%
**Full clinical recovery****	91.52%
Clinical intercurrences	19.61%
Scleral instability + dyscoria	11.77%
Corneal dystrophy	3,92%
Hyfema	3,92%

Supplementary Table S3. Clinical details of the procedure.

*n=75; **n=59 (animals followed for ≥ 14 days after surgery); full clinical recovery: clear optic media, trophic eye globe and normal IOP.

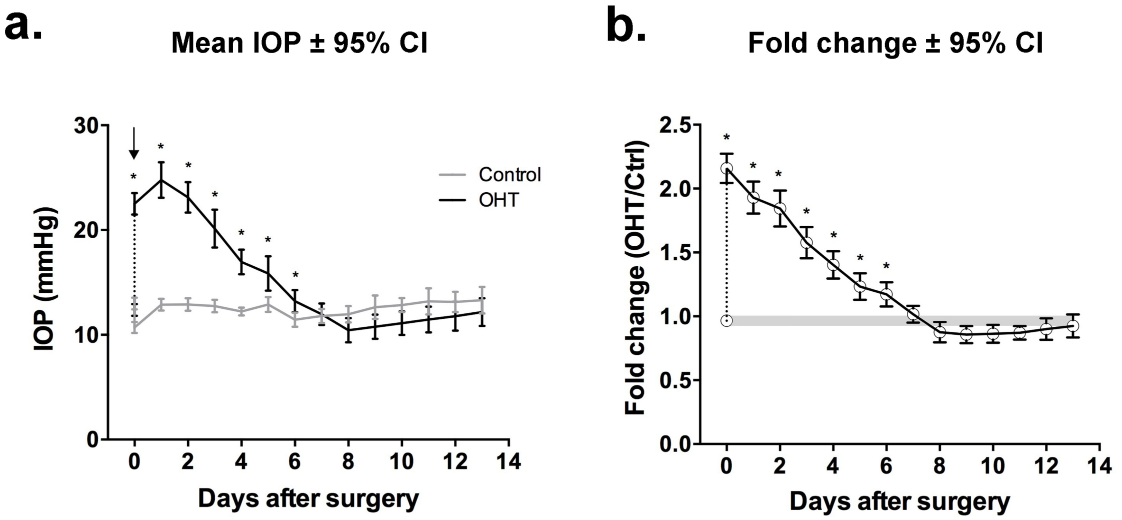

Supplementary Figure S1. IOP dynamics after LPC induction of OHT. (a) Mean IOP ± 95% CI. **(b)** Fold change of IOP (OHT/contralateral control eye or Ctrl) ± 95% CI. Statistical analysis of (a) and (b): multiple t test, with statistical significance determined using the Holm-Sidak method; (*)p<0.05. In (a), comparison was between OHT and control groups for each day after surgery; in (b), comparison was between the post-op ratios (OHT/Ctrl) and the pre-op one (here represented as the gray strip). N = 17 – 69 (see Supplementary Table S2). Vertical downward arrows: LPC surgery.

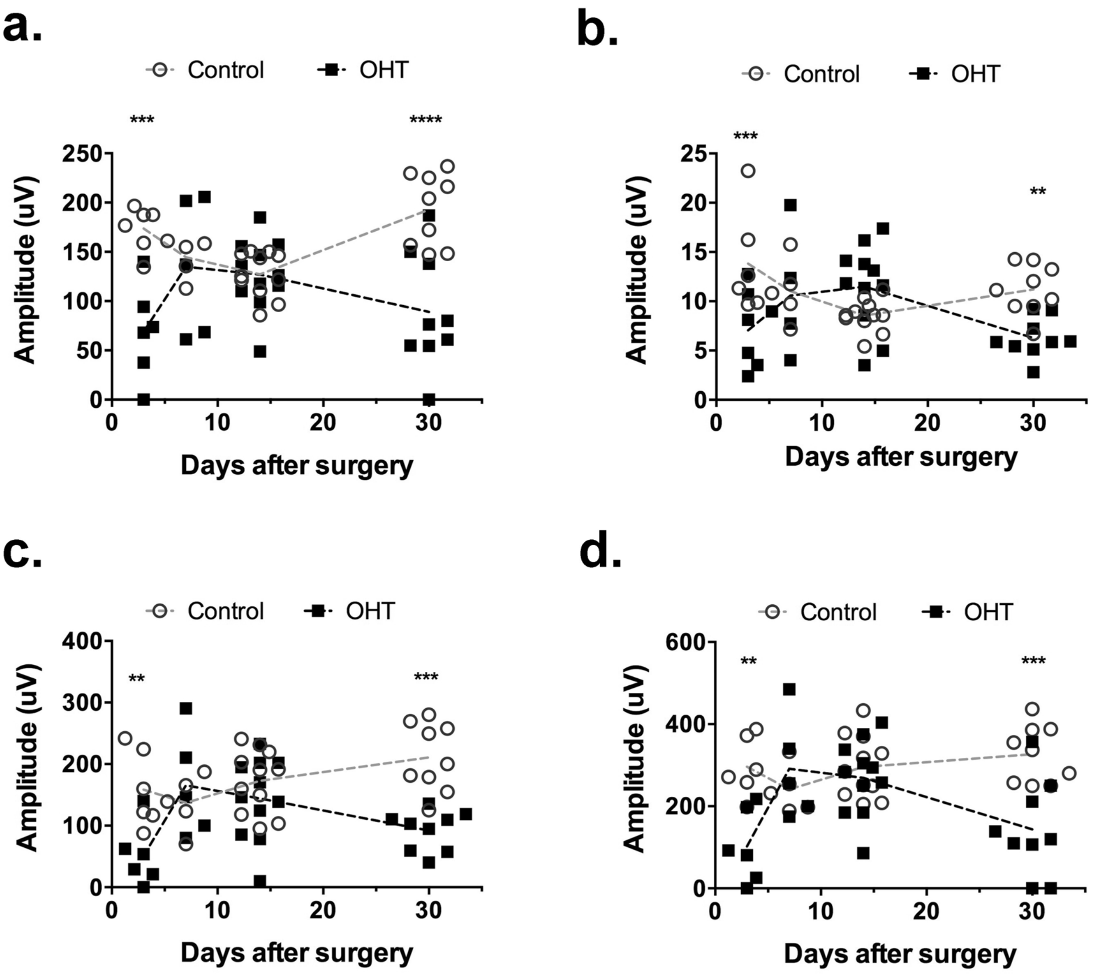

Supplementary Figure S2. Flash ERG (FERG) amplitude over time after OHT induction with LPC model. FERG, examination technique used to study the outer retinal function, was acquired in both photopic and scotopic conditions. **(a)** Photopic b-wave amplitude, a measure of cone-related bipolar cells function; **(b)** Photopic flicker amplitude, parameter that isolates the bioelectric function of cones; **(c)** scotopic a-wave, negative deflection that highlight the function of photoreceptors, mostly rods; **(d)** scotopic b-wave, positive deflection that mostly indicates the function of rods-related bipolar cells. The data points out a pattern of amplitude decrease in the four parameters analyzed early during the clinical evolution of the animals, at a time point when IOP is still elevated, and later one month after surgery. After IOP return to baseline levels 7 days after surgery, the outer retinal function becomes statistically similar to the contralateral control eyes, which is also seen after two weeks. Statistical analysis with two-way ANOVA with Sidak's multiple comparisons post hoc test; (*)$p<0.05$, (**)$p<0.01$, (***)$p<0.001$, (****)$p<0.0001$.
